# The associations of depressive symptoms and perceived stress with arterial health in adolescents

**DOI:** 10.14814/phy2.15986

**Published:** 2024-03-22

**Authors:** Emmi Toivonen, Earric Lee, Marja H. Leppänen, Tomi Laitinen, Mika Kähönen, Timo A. Lakka, Eero A. Haapala

**Affiliations:** ^1^ Faculty of Sports and Health Sciences University of Jyväskylä Jyväskylä Finland; ^2^ Institut de Cardiologie de Montréal Montréal QC Canada; ^3^ École de kinésiologie et des sciences de l’activité physique Université de Montréal Montréal QC Canada; ^4^ Institute of Biomedicine, University of Eastern Finland Kuopio Campus Kuopio Finland; ^5^ Department of Clinical Physiology and Nuclear Imaging University of Eastern Finland and Kuopio University Hospital Kuopio Finland; ^6^ Department of Clinical Physiology, Tampere University Hospital and Faculty of Medicine and Health Technology Tampere University Tampere Finland; ^7^ Foundation for Research in Health Exercise and Nutrition Kuopio Research Institute of Exercise Medicine Kuopio Finland

**Keywords:** adolescent, arterial stiffness, cardiovascular risk factors, depression, perceived stress

## Abstract

Cardiovascular and mental diseases are among the most important global health problems, but little is known on the associations between mental and arterial health in adolescents. Therefore, we investigated the associations of arterial health with depressive symptoms and perceived stress in adolescents. A total of 277 adolescents, 151 boys, 126 girls, aged 15–17 years participated in the study. Depressive symptoms were assessed using the Beck Depression Inventory and perceived stress by the Cohen Perceived Stress Scale. Arterial health was assessed by measures from carotid ultrasonography (carotid intima‐media thickness, Young's Elastic Modulus, carotid artery distensibility, stiffness index), impedance cardiography (pulse wave velocity, cardio‐ankle vascular index), and pulse contour analysis (reflection index, stiffness index). The data were analyzed using linear regression models adjusted for age and sex. Depressive symptoms or perceived stress were not associated with indices of arterial health in the whole study group (*β* = −0.08 to 0.09, *p* > 0.05), in boys (*β* = −0.13 to 0.10, *p* > 0.05) or in girls (standardized regression coefficient *β* = −0.16 to 0.08, *p* > 0.05). We found no associations of depressive symptoms and perceived stress with arterial health in adolescents. These observations suggest that the association between mental and arterial health problems develop in later life.

## 1 INTRODUCTION

Cardiovascular diseases (CVD) have a significant influence on morbidity and mortality. Global prevalence of CVD and cardiovascular mortality has also increased substantially during the past two decades (Roth et al., 2021). The pathophysiological origins of CVD, including arterial stiffening and atherosclerotic lesion formation characterized by fractures in elastin, collagen overproduction, and accumulation of lipids and fibrous tissue to the artery wall, could begin as early as adolescence (Agbaje, 2023; Flore et al., 2015; Vlachopoulos et al., 2010). During this period, mental health problems affect about 13% of adolescents globally (Polanczyk et al., 2015), with depression being the main reason for mental ill‐being (Whiteford et al., 2013). Adolescents are more vulnerable to stress, and stressors have a greater impact on brain function on adolescence compared to adulthood (Eiland & Romeo, 2013; Pervanidou & Chrousos, 2012). However, little is known about the associations of depression and stress with cardiovascular risk in adolescence, although it has been found in adulthood (Hare et al., 2014; Kivimaki & Steptoe, 2018; Park et al., 2023). Identifying potential risk factors and preventing CVD early in life will lower its associated burden on individuals, public healthcare systems, and the economy (Laslett et al., 2012; Leal et al., 2006).

Greater depressive symptoms have previously been associated with a higher pulse wave velocity (PWV) (Dietz & Matthews, 2011) and a lower ankle‐brachial index (ABI) in adolescents (Tonhajzerova et al., 2022). However, conflicting reports exist. Some previous studies have reported weak and statistically insignificant associations of depressive symptoms with brachial‐ankle‐PWV and cardio‐ankle vascular index (CAVI) (Tonhajzerova et al., 2022), carotid intima‐media thickness (cIMT), carotid artery distensibility, carotid artery compliance, and carotid artery elastic modulus (Gross et al., 2018) in adolescents. Most of these previous studies have investigated the associations between depressive symptoms and arterial health, and only one study explored the role of perceived stress as a risk factor for impaired arterial health (Olive et al., 2020). The authors reported an insignificant association between perceived stress and PWV. With the exception of the work performed by Gross et al. (2018), the aforementioned studies assessed arterial health with only a small number of indices and did not consider pubertal status as a covariate.

The pathophysiological mechanisms that may explain depression‐ and stress‐induced changes in arterial stiffness and atherosclerosis leading to impaired arterial health include increased low‐grade inflammation, decreased heart rate variability (HRV), elevated sympathoadrenal and hypothalamic–pituitary–adrenal axis (HPA‐axis) activity, and endothelial dysfunction (Nemeroff & Goldschmidt‐Clermont, 2012). Furthermore, increased cortisol concentration could also contribute to arterial stiffening which may lead to hypertension (Connell et al., 1987; Esler et al., 2008; Kelly et al., 1998; Pirpiris et al., 1993; Ushakov et al., 2016) and endothelial dysfunction (Black & Garbutt, 2002; García‐Bueno et al., 2008; Toda & Nakanishi‐Toda, 2011). Associations between mental and arterial health and the underlying pathophysiology may differ between boys and girls due to the different rate of maturation between sexes (Abreu & Kaiser, 2016; Brix et al., 2018), as pubertal development affects hormonal system and adipose tissue growth (Bergeron et al., 2015; Dunkel, 2009). These associations have yet to be thoroughly investigated separately for both sexes.

The associations between mental and arterial health in adolescents have yet to be fully elucidated. By improving our understanding in the domain, we may be able to detect early signs of possible cardiovascular risk in adolescence due to mental ill‐being, which could have important implications for adulthood. Therefore, we explored the associations of depressive symptoms and perceived stress with several indices of arterial health including systolic blood pressure, cIMT, Young's Elastic Modulus, carotid artery distensibility, and stiffness index assessed by a carotid ultrasound, PWV and CAVI assessed using impedance cardiography, and stiffness index and reflection index assessed using pulse contour analyses to attain a more comprehensive perspective.

## 2 METHODS

The present cross‐sectional study is based on the 8‐year follow‐up data from The Physical Activity and Nutrition in Children (PANIC) study. It is a controlled lifestyle intervention study in a population sample of children from the city of Kuopio, Finland (Eloranta et al., 2011). The Research Ethics Committee of the Hospital District of Northern Savo approved the study protocol in 2006 (Statement 69/2006) and in 2015 (Statement 422/2015). All adolescents and their parents gave written informed consent. The PANIC Study has been carried out in accordance with the principles of the Declaration of Helsinki as revised in 2008. The study was registered at Clinicaltrials.gov (NCT01803776).

736 children aged 6–8 years from primary schools of Kuopio were invited to participate in the baseline examination between 2007 and 2009. Inclusion criteria for participation were apparently healthy boy or girl, 6–9 years of age at baseline, living in the city of Kuopio, Finland. The exclusion criteria included any physical disability that could hamper participation in the intervention or no time or motivation to attend the study. A total of 512 children, who represented 70% of those invited, participated in the baseline examinations. 8‐year follow‐up examinations were performed between 2016 and 2017, and a total of 277 (N) adolescents (151 boys and 126 girls) aged 15–17 attended. We performed the main statistical analyses using the largest number of participants available for each analysis, and the number of participants in the main analyses varied from 206 to 265 (108–141 in boys, 98–125 in girls) due to missing data. In the complete case analyses, there was a total of 101 boys and 87 girls with complete data on all variables of interest.

### 2.1 Assessment on arterial health

Systolic (SBP) and diastolic blood pressure (DBP) were measured from the right arm using the Heine Gamma G7® aneroid sphygmomanometer (Heine Optotechnik) to the accuracy of 2 mm Hg. The measurement protocol included a 5 min seated resting period followed by three measurements with 2 min intervals in between. The average of all three values was used for both SBP and DBP.

For the assessment of cIMT and elasticity of the left common carotid artery, carotid ultrasound imaging was performed utilizing the Acuson Sequoia 512 Ultrasound Mainframe® (Acuson, Mountain View, CA, USA) with a 14.0 MHz linear array transducer using a standardized protocol (Raitakari et al., 2003). The sonographers analyzed the ultrasound scans offline from the digitally stored images. Three measurements of the far wall at end‐diastole were taken to derive maximal cIMT. To assess carotid artery elasticity, the diameter of the common carotid artery at end‐diastole and end‐systole was measured at least twice. In addition, the sonographer measured systolic (SBP) and DBP from the brachial artery just before and directly after the ultrasound scans. The means of the end‐diastolic and end‐systolic diameters as well as SBP and DBP values were used to calculate arterial elasticity indices. Carotid artery distensibility (CAD) was calculated as:

$$\frac{\left(\text{systolic}-\text{diastolic diameter}\right)÷\text{diastolic diameter}}{\left(\text{SBP}-\text{DBP}\right)}$$

Young's Elastic Modulus as:

$$\frac{\left(\left[\text{SBP}-\text{DBP}\right]\times \text{diastolic diameter}\right)}{\left(\left[\text{systolic}-\text{diastolic diameter}\right]÷\text{IMT}\right)}$$

and Stiffness Index (SI) as:

$$\text{ln}\frac{\left(\text{SBP}÷\text{DBP}\right)}{\left[\left(\text{systolic}-\text{diastolic diameter}\right)÷\text{diastolic diameter}\right]}$$

These measurements and analyses were performed at Department of Clinical Physiology and Nuclear Medicine, Kuopio University Hospital by trained sonographers (Raitakari et al., 2003). Pulse wave velocity (PWV) was measured by impedance cardiography using the Circmon® 419 B202 impedance cardiography device (JR Medical Ltd, Saku Vald, 420 Estonia), using previously documented methods (Koivistoinen et al., 2007). Cardio‐ankle vascular index (CAVI) was measured by recording the distance from the level of the aortic valve brachial level to the ankle and the time delay between the closing of the aortic valve to the detected change in arterial pressure wave at the set point. Details have been published elsewhere (Yambe et al., 2004).

Arterial stiffness index (SIpca) and reflection index (RIpca) were assessed by pulse contour analysis using non‐invasive finger photoplethysmography with the PulseTrace PCA2 device (MicroMedical, Gillingham, Kent, United Kingdom) as has been described previously (Veijalainen et al., 2011, 2013). SI was assessed in a supine position prior to the exercise test in a test laboratory at stable room temperature (20–22°C) after a 15‐min rest. SI was calculated by dividing body height by time between the first (systolic) peak and the second (diastolic) peak of the pulse contour and was expressed in meters per second. All the pulse contour data were visually evaluated, and the data were excluded from the analysis if an extra peak could be seen between the systolic and diastolic peak, or if the device had measured the indexes incorrectly or if the same child had considerable differences in the pulse contour values (Veijalainen et al., 2011). Reflection index was calculated as the percentage of the height of the second peak from the height of the first peak (Veijalainen et al., 2011).

### 2.2 Assessment of depressive symptoms and perceived stress

Depressive symptoms were assessed by the Beck's Depression Inventory that measures characteristic attitudes and symptoms of depression. It contains 21 self‐reportable questions from scale 0 to 3, with a maximum possible point total of 63 (Beck et al., 1961). Beck's Depression Inventory is a suitable tool for assessing depressive symptoms in adolescents (Bennet et al., 1997; Stockings et al., 2015). Perceived stress was assessed by the Cohen Perceived Stress Scale (Cohen et al., 1983). The Finnish version (PSS‐10) contains 10 questions from scale 0 to 4, for a maximum of 40 points. The Cohen Perceived Stress Scale is effective in detecting perceived stress in adolescents (White, 2014). In both of these scales, higher scores indicate greater difficulties in mental health.

### 2.3 Other assessments

Body weight was measured using a calibrated InBody 720® bioelectrical impedance device (Biospace, Seoul, South Korea). Height was measured using a wall‐mounted stadiometer without shoes (Leppänen et al., 2019). BMI was calculated by dividing body weight (kg) by height (m) squared, and BMI‐SDS was obtained using Finnish references (Saari et al., 2011). Visceral adipose tissue was measured using the Lunar® dual‐energy x‐ray absorptiometry device (GE Medical Systems, Madison, Wisconsin, USA) (Lakka et al., 2020).

A research physician assessed pubertal status using the 5‐stage scale described by Tanner (Marshall & Tanner, 1969, 1970). We used breast development in girls and testicular volume in boys to assess pubertal status. Parental education was categorized based on either ongoing or completed education of mothers and fathers (vocational school or less, polytechnic, or university degree) (Eloranta et al., 2011). Household income was categorized as ≤30,000, 30,001–60,000 and >60,000 €/year (Eloranta et al., 2011). The highest level of education and household income reported by either parent were used in the analyses.

### 2.4 Statistical analyses

All data analyses were performed using the SPSS Statistics software, version 28.0 (IBM Corporation, Armonk, NY, USA). Differences in characteristics between boys and girls were investigated using the *t*‐test for normally distributed continuous variables, the Mann–Whitney *U*‐test for continuous variables with skewed distributions and the chi‐squared test for categorical variables. The associations of depressive symptoms and perceived stress with cardiovascular risk factors were investigated using the multivariate linear regression analysis. All the analyses were performed for the whole study group adjusted for age and sex, and also separately for boys and girls adjusted for age. The data were further adjusted for pubertal status, BMI‐SDS, visceral adipose tissue, household income or parental education, which were entered into the models separately. We performed the analyses using the highest number of participants in each analysis. We also performed sensitivity analyses using complete sets of data for all variables of interest. *p*‐values lower than 0.05 were considered statistically significant.

## 3 RESULTS

### 3.1 Characteristics of participants

Boys were taller and heavier and had more visceral adipose tissue than girls (Table 1). Boys also had less advanced pubertal stage and had higher SBP, cIMT, YEM, SI, RIpca and SIpca and lower CAD than girls.

**TABLE 1:** Characteristics of participants.

	Boys (*n = 109–151*)	Girls (*n = 99–126*)	*p* for difference
Age (years)	15.85 (0.46) (*151*)	15.75 (0.39) (*126*)	0.064
Weight (kg)	65.8 (15.1) (*151*)	57.9 (9.1) (*125*)	**<0.001**
Height (cm)	176.6 (7.4) (*151*)	165.7 (5.8) (*125*)	**<0.001**
BMI‐SDS	−0.14 (1.11) (*151*)	0.05 (0.89) (*125*)	0.115
Visceral adipose tissue (g)	209.3 (337.5) (*138*)	121.3 (149.4) (*124*)	**0.003**
Pubertal status**			
3	13.4 (*17*)	3.5 (*4*)	**<0.001**
4	61.4 (*78*)	53.5 (*61*)	
5	25.2 (*32*)	43.0 (*49*)	
Household income (€/y)**			
≤30,000	10.9 (*14*)	7.9 (*8*)	0.102
30,001–60,000	18.8 (*24*)	29.8 (*34*)	
>60,000	70.3 (*90*)	63.2 (*72*)	
Parental education**			
Vocational school	13.8 (*18*)	14.5 (*17*)	**0.012**
Polytechnic	32.3 (*42*)	49.6 (*58*)	
University	53.8 (*70*)	35.9 (*42*)	
Beck's Depression Inventory score* (0 to 63)	1.0 (1.0–2.5) (*137*)	2.0 (0.0–7.0) (*121*)	**<0.001**
Cohen Perceived Stress Scale score (0 to 40)	11.0 (5.5) (*141*)	15.1 (5.7) (*125*)	**<0.001**
Systolic blood pressure (mmHg)	115.7 (10.9) (*151*)	110.4 (9.1) (*124*)	**<0.001**
Carotid intima‐media thickness (mm)	0.45 (0.06) (*140*)	0.43 (0.05) (*125*)	**0.002**
Young's elastic modulus*	169.5 (138.6–206.8) (*140*)	145.6 (125.3–184.8) (*125*)	**<0.001**
Carotid artery distensibility (%/10 mmHg)	2.68 (0.71) (*140*)	2.90 (0.70) (*125*)	**0.013**
Carotid artery stiffness index	4.42 (1.16) (*140*)	4.14 (0.93) (*100*)	**0.029**
Pulse wave velocity (m/s)*	5.65 (5.38–6.10) (*117*)	5.73 (5.46–6.10) (*104*)	0.381
Cardio‐ankle vascular index *	6.05 (5.38–7.18) (*109*)	6.35 (5.60–7.10) (*99*)	0.240
Reflection index from pulse contour analysis (%)	58.1 (14.7) (*132*)	46.9 (12.9) (*100*)	**<0.001**
Stiffness index from pulse contour analysis (m/s)*	5.7 (5.4–6.1) (*132*)	5.2 (4.9–5.4) (*100*)	**<0.001**

*Note*: The data are means (standard deviations), *medians (interquartile ranges) or **percentages. The *p*‐values are from the *t*‐test for continuous variables with normal distributions, the Mann Whitney *U*‐test for continuous variables with skewed distributions and the chi‐squared test for categorical variables. Abbreviations: BMI, body mass index; BMI‐SDS, body mass index standard deviation score; *p*‐values for statistically significant differences (*p* < 0.05) are in bold.

### 3.2 Associations of depressive symptoms and perceived stress with indices of arterial health

Depressive symptoms or perceived stress were not associated with measures of arterial health after adjustment for age and sex (Tables 2 and 3). Further adjustments had no effect on the magnitude of these associations. The associations of depressive symptoms and perceived stress with indices of arterial health among participants with complete data on all variables of interest are presented in supplementary material and Tables S1 and S2.

**TABLE 2:** Associations of depressive symptoms with measures of arterial health.

	All (*n* = 206–257)			Boys (*n* = 108–137)			Girls (*n* = 98–121)		
	*β* (95% CI)	Beta	*p*‐value	*β* (95% CI)	Beta	*p*‐value	*β* (95% CI)	Beta	*p*‐value
Systolic blood pressure (mmHg)	−0.04 (−0.09;‐0.02)	−0.08	0.217	0.00 (−0.05;0.05)	−0.01	0.931	−0.10 (−0.22;0.02)	−0.16	0.095
Carotid intima‐media thickness (mm)	−0.19 (−10.22;9.83)	0.00	0.970	2.85 (−5.71;11.41)	0.06	0.511	−5.50 (−26.71;15.71)	−0.05	0.608
Young's elastic modulus	0.00 (−0.01;0.01)	0.00	0.944	0.00 (−0.01;0.01)	−0.02	0.860	0.000 (−0.02;0.02)	0.00	0.985
Carotid artery distensibility (%/10 mmHg)	−0.12 (−0.94;0.70)	−0.02	0.771	0.12 (−0.64;0.88)	0.03	0.749	−0.39 (−1.91;1.14)	−0.05	0.618
Carotid artery stiffness index	0.07 (−0.48;0.61)	0.02	0.813	−0.07 (−0.53;0.40)	−0.03	0.770	0.34 (−0.81;1.50)	0.05	0.558
Pulse wave velocity (m/s)	0.15 (−0.91;1.20)	0.02	0.782	0.37 (−0.59;1.34)	0.07	0.446	−0.18 (−2.19;1.83)	−0.02	0.859
Cardio‐ankle vascular index	−0.11 (−0.54;0.31)	−0.04	0.596	0.12 (−0.54;0.30)	−0.06	0.568	−0.08 (−0.82;0.65)	−0.02	0.826
Reflection index from pulse contour analysis (%)	0.00 (−0.05;0.04)	−0.02	0.821	0.02 (−0.02;0.06)	0.10	0.259	−0.06 (−0.16;0.04)	−0.12	0.232
Stiffness index from pulse contour analysis (m/s)	−0.52 (−1,66;0.62)	−0.07	0.370	−0.76 (−1.18;0.24)	−0.13	0.133	−0.09 (−2.64;2.46)	−0.01	0.945

*Note*: Data are unstandardized regression coefficients and their 95% confidence intervals (β), standardized regression coefficients (Beta) and *p*‐values from linear regression models adjusted for age and sex in all participants and for age in sex‐stratified analyses.

**TABLE 3:** Associations of perceived stress with measures of arterial health.

	All (*n* = 206–265)			Boys (*n* = 109–141)			Girls (*n* = 99–125)		
	*β* (95% CI)	Beta	*p*‐value	*β* (95% CI)	Beta	*p*‐value	*β* (95% CI)	Beta	*p*‐value
Systolic blood pressure (mmHg)	−0.04 (−0.11;0.02)	−0.08	0.194	−0.04 (−0.13;0.04)	−0.09	0.305	−0.05 (−0.16;0.07)	−0.07	0.427
Carotid intima‐media thickness (mm)	4.06 (−7.85;15.98)	0.04	0.503	2.32 (−12.51;17.15)	0.03	0.757	7.14 (−12.14:27.43)	0.06	0.487
Young's elastic modulus	0.00 (−0.02;0.01)	−0.01	0.868	−0.01 (−0.03;0.01)	−0.06	0.510	0.01 (−0.02;0.03)	0.05	0.551
Carotid artery distensibility (%/10 mmHg)	0.09 (−0.87;1.06)	0.01	0.848	0.38 (−0.93;1.69)	0.05	0.567	−0.24 (−1.69;1.21)	−0.03	0.748
Carotid artery stiffness index	−0.04 (−0.68;0.61)	−0.01	0.916	−0.15 (−0.95;0.66)	−0.03	0.718	0.16 (−0.94;1.27)	0.03	0.768
Pulse wave velocity (m/s)	0.20 (−1.00;1.39)	0.02	0.738	0.78 (−0.80;2.37)	0.09	0.330	−0.49 (−2.14;1.35)	−0.05	0.600
Cardio‐ankle vascular index	−0.14 (−0.61;0.33)	−0.04	0.559	−0.05 (−0.73;0.63)	−0.02	0.881	−0.21 (−0.89;0.47)	−0.06	0.543
Reflection index from pulse contour analysis (%)	0.04 (−0.02;0.09)	0.09	0.195	0.03 (−0.03;0.10)	0.09	0.317	0.04 (−0.05;0.13)	0.08	0.410
Stiffness index from pulse contour analysis (m/s)	−0.76 (−2.16;0.64)	−0.08	0.285	−0.83 (−2.56;0.91)	−0.08	0.348	−0.63 (−3.06;1.80)	−0.05	0.607

*Note*: Data are unstandardized regression coefficients and their 95% confidence intervals (β), standardized regression coefficients (Beta) and *p*‐values from linear regression models adjusted for age and sex in all participants and for age in sex‐stratified analyses.

## 4 DISCUSSION

We found no associations of depressive symptoms and perceived stress with measures of arterial health in adolescent boys and girls. These observations suggest that depressive symptoms and perceived stress are not cardiovascular risk factors in a general population of Finnish adolescents.

Some previous studies have shown an inverse association between depressive symptoms and arterial health indices in adolescents (Belem da Silva et al., 2019; Dietz & Matthews, 2011; Tonhajzerova et al., 2022), which is contrary to the present findings. One explanation for this inconsistency may be that these earlier studies included participants with more severe depressive symptoms (Belem da Silva et al., 2019; Dietz & Matthews, 2011; Tonhajzerova et al., 2022), whereas our study sample consisted of generally healthy adolescents. Moreover, Belem da Silva et al. (2019) used a longitudinal approach with a duration of 3 or 7 years using a relatively larger population (*n* = 4336 with complete mental health data). As such, there is a likelihood that a relatively longer sustenance of chronic depressive symptoms could negatively affect arterial health.

We found no statistically significant association between depressive symptoms and several indices of arterial health. Our observations on the weak associations between depressive symptoms and arterial health are partially in line with the results by Tonhajzerova et al. (2022), who found no relationship between depressive symptoms to PWV and CAVI. However, higher depressive symptoms were shown to be associated with lower, albeit normal ABI, indicating poorer arterial health. While the reason for the stronger association between depressive symptoms and ABI compared to that of PWV and CAVI is unclear, the evidence suggests that ABI is a useful measure for detecting peripheral arterial problems. Low values of ABI could also be caused by reduced physical activity or autonomic dysregulation that are typical with severely depressed (Nemeroff & Goldschmidt‐Clermont, 2012; Parsons et al., 2016) and, therefore, be more sensitive to depressive symptoms than PWV and CAVI. Moreover, the aforementioned study (Tonhajzerova et al., 2022) did not account for important covariates such as sex and height. ABI has been shown to be sensitive to variations in age, sex, height, and measurement order than PWV and/or CAVI (Aboyans et al., 2012).

In line with the only other available study on the association of perceived stress and PWV in adolescents (Olive et al., 2020), we only found rather weak associations between perceived stress PWV, and other indices of arterial health. These findings in adolescents differ from those reported in adults, suggesting that increased perceived stress may lead to increased arterial stiffness (Logan et al., 2012, 2020; Mauss et al., 2021). Although the evidence remains somewhat inconclusive, adult‐based studies seem to suggest that perceived stress may indeed have an effect on arterial stiffness (Bugajska et al., 2008; Wiernik et al., 2016). Nevertheless, there may be a few possible reasons for the insignificant results between mental and arterial health in our study. Impaired arterial health may require a longer, and more severe exposure to depressive symptoms and perceived stress in order for it to be reflected. The relatively healthy mental and physical (Randrianarisoa et al., 2015; The European Society of Hypertension (ESH) and of the European Society of Cardiology (ESC), 2013; Salonen & Salonen, 1993) characteristics of our participants may also have contributed additionally to that. As such, caution must be paid when comparing the present results to other similar populations with a greater prevalence of depression or perceived stress. Adolescence is a challenging period of life, as various physiological systems undergo perturbations and modifications. For instance, cIMT increases during maturation and with increasing age (Doyon et al., 2013; Jourdan et al., 2005; Randrianarisoa et al., 2015; Torkar et al., 2020). As such, increased cIMT might not solely reflect atherosclerotic or pathological changes, but maturation‐related changes as well.

The strengths of the present study include a comprehensive, reproducible and valid assessment of arterial health. We used well‐validated instruments for depression symptoms and perceived stress (Stockings et al., 2015; White, 2014), and accounted for several potential confounding factors. However, our study included a sample of relatively healthy adolescents with low levels of depressive symptoms and perceived stress, decreasing the generalisability of our results to adolescents with clinical mental disorders. Thus, low levels of depressive symptoms and perceived stress may reduce the magnitude of the associations between mental and arterial health in our study population. Moreover, a cross‐sectional design precludes causal interpretations.

In conclusion, depressive symptoms and perceived stress were not associated with indices of arterial health in adolescents. Therefore, it remains uncertain whether depressive symptoms or perceived stress increase cardiovascular risk already in adolescence. More studies investigating whether depressive symptoms and perceived stress are related to cardiovascular stress responses in adolescents are needed. More crucially, the longitudinal relationship between arterial health, and depressive symptoms and perceived stress in a similar population group are warranted. This will provide further insight as to whether these associations would carry on into ones' early adulthood.

## AUTHOR CONTRIBUTIONS

*Conceptualization*: Emmi Toivonen, Earric Lee, Eero A. Haapala; *Methodology*: Marja H. Leppänen, Mika Kähönen, Tomi Laitinen, Timo A. Lakka, Eero A. Haapala; *Formal analysis and investigation*: Emmi Toivonen; *Writing‐original draft preparation*: Emmi Toivonen; *Writing‐review and editing*: Earric Lee, Marja H. Leppänen, Tomi Laitinen, Mika Kähönen, Timo A. Lakka, Eero A. Haapala; *Funding acquisition*: Timo A. Lakka, Eero A. Haapala; *Resources*: Tomi Laitinen, Mika Kähönen, Timo A. Lakka; *Supervision*: Timo A. Lakka, Eero A. Haapala. All authors approved the final version of the manuscript.

## AKNOWLEDGEMENTS

The PANIC study has been supported financially by grants from the Research Council of Finland, Ministry of Education and Culture of Finland, Ministry of Social Affairs and Health of Finland, Research Committee of the Kuopio University Hospital Catchment Area (State Research Funding), Finnish Innovation Fund Sitra, Social Insurance Institution of Finland, Finnish Cultural Foundation, Foundation for Pediatric Research, Diabetes Research Foundation in Finland, Finnish Foundation for Cardiovascular Research, Juho Vainio Foundation, Paavo Nurmi Foundation, Yrjö Jahnsson Foundation, and the city of Kuopio.

## CONFLICT OF INTEREST STATEMENT

The authors declare they have no competing interests.

## ETHICS STATEMENT

The Research Ethics Committee of the Hospital District of Northern Savo approved the study protocol in 2006 (Statement 69/2006) and in 2015 (Statement 422/2015). All adolescents and their parents gave written informed consent. The PANIC Study has been carried out in accordance with the principles of the Declaration of Helsinki as revised in 2008.

## Supporting information


Data S1.

## Data Availability

The data are not publicly available due to research ethical reasons and because the owner of the data is the University of Eastern Finland and not the research group. However, the corresponding author can provide further information on the PANIC study and the PANIC data on a reasonable request.
