# Supplementary material for: The associations of depressive symptoms and perceived stress with arterial health in adolescents
Source: Physiol Rep. 2024 Mar 22;12(6):e15986. doi: 10.14814/phy2.15986 (PMC10959692; doi:10.14814/phy2.15986)
Supplement: Supplementary file 1 — Data S1. [file PHY2-12-e15986-s001.docx]

**SUPPLEMENTARY MATERIAL**

**The Associations of Depressive Symptoms and Perceived Stress with Arterial Health in Adolescents**

Emmi Toivonen^1^, Earric Lee^1^, Marja H. Leppänen^2^, Tomi Laitinen^3^, Mika Kähönen^4^, Timo A. Lakka^2,3,5^, Eero A. Haapala^1,2^

^1^Faculty of Sports and Health Sciences, University of Jyväskylä, Jyväskylä, Finland; ^2^Institute of Biomedicine, University of Eastern Finland, Kuopio Campus, Finland; ^3^Department of Clinical Physiology and Nuclear Imaging, University of Eastern Finland and Kuopio University Hospital, Kuopio, Finland; ^4^Department of Clinical Physiology, Tampere University Hospital and Faculty of Medicine and Health Technology, Tampere University, Tampere, Finland; ^5^Foundation for Research in Health Exercise and Nutrition, Kuopio Research Institute of Exercise Medicine, Kuopio, Finland.

Address correspondence to: Dr Eero Haapala, PhD, Faculty of Sport and Health Sciences, University of Jyväskylä, Jyväskylä, Finland, email: [eero.a.haapala@jyu.fi](mailto:eero.a.haapala@jyu.fi)

| **Supplementary Table S1**. Associations of depressive symptoms with arterial health in 101 boys and 87 girls with complete data on depressive symptoms, perceived stress and measures of arterial health. | | | | | | |
| --- | --- | --- | --- | --- | --- | --- |
|  | Boys (n=101) | | | Girls (n=87) | | |
|  | ß (95% CI) | Beta | P-value | ß (95% CI) | Beta | P-value |
| Systolic blood pressure (mmHg) | 0.002 (-0.055;0.059) | 0.007 | 0.949 | -0.054 (-0.193;0.085) | -0.085 | 0.442 |
| Carotid intima-media thickness (mm) | 7.142 (-3.267;17.551) | 0.136 | 0.176 | -8.565 (-35.537;18.407) | -0.069 | 0.529 |
| Young's elastic modulus | 0.001 (-0.010;0.012) | 0.015 | 0.881 | 0.001 (-0.029;0.031) | 0.007 | 0.948 |
| Carotid artery distensibility (%/10mmHg) | 0.189 (-0.785;1.162) | 0.039 | 0.701 | -0.636 (-2.596;1.324) | -0.071 | 0.520 |
| Carotid artery stiffness index | -0.118 (-0.669;0.432) | -0.043 | 0.670 | 0.534 (-0.865;1.933) | 0.083 | 0.450 |
| Pulse wave velocity (m/s) | -0.824 (-2.030;0.382) | -0.137 | 0.178 | -0.231 (-2.325;1.862) | -0.024 | 0.827 |
| Cardio-ankle vascular index | -0.133 (-0.571;0.306) | -0.060 | 0.550 | 0.022 (-0.719;0.763) | 0.007 | 0.953 |
| Reflection index from pulse contour analysis (%) | 0.029 (-0.015;0.074) | 0.130 | 0.197 | -0.085 (-0.200;0.029) | -0.161 | 0.140 |
| Stiffness index from pulse contour analysis (m/s) | -0.582 (-1.744;0.580) | -0.100 | 0.323 | -0.336 (-3.091;2.420) | -0.026 | 0.809 |

Data are unstandardised regression coefficients and their 95% confidence intervals (ß), standardised regression coefficients (Beta) and p-values. Data were adjusted for age.

|  | | |  |  |  |  |
| --- | --- | --- | --- | --- | --- | --- |
| **Supplementary Table S2**. Associations of perceived stress with arterial health in 101 boys and 87 girls with complete data on depressive symptoms, perceived stress and measures of arterial health. | | | | | | |
|  | Boys (n=101) | | | Girls (n=87) | | |
|  | ß (95% CI) | Beta | P-value | ß (95% CI) | Beta | P-value |
| Systolic blood pressure (mmHg) | -0.064 (-0.154;0.025) | -0.143 | 0.157 | -0.048 (-0.179;0.084) | -0.079 | 0.475 |
| Carotid intima-media thickness (mm) | 5.455 (-11.196;22.107) | 0.066 | 0.517 | 5.575 (-19.935;31.085) | 0.047 | 0.665 |
| Young's elastic modulus | -0.002 (-0.020;0.016) | -0.019 | 0.849 | -0.005 (-0.033;0.024) | -0.036 | 0.741 |
| Carotid artery distensibility (%/10mmHg) | 0.235 (-1.174;1.643) | 0.033 | 0.742 | 0.477 (-1.376;2.330) | 0.056 | 0.610 |
| Carotid artery stiffness index | 0.024 (-0.852;0.899) | 0.005 | 0.957 | -0.339 (-1.663;0.985) | -0.055 | 0.612 |
| Pulse wave velocity (m/s) | -0.755 (-2.682;1.173) | -0.079 | 0.439 | -0.582 (-2.556;1.392) | -0.064 | 0.559 |
| Cardio-ankle vascular index | -0.116 (-0.813;0.580) | -0.033 | 0.741 | -0.185 (-0.884;0.514) | -0.058 | 0.600 |
| Reflection index from pulse contour analysis (%) | 0.034 (-0.037;0.105) | 0.096 | 0.343 | 0.033 (-0.076;0.141) | 0.065 | 0.554 |
| Stiffness index from pulse contour analysis (m/s) | -0.184 (-2.038;1.671) | -0.020 | 0.844 | -0.597 (-3.198;2.004) | -0.050 | 0.649 |

Data are unstandardised regression coefficients and their 95% confidence intervals (ß), standardised regression coefficients (Beta) and p-values. Data were adjusted for age.
